# Rice Transcription Factor OsWRKY55 Is Involved in the Drought Response and Regulation of Plant Growth

**DOI:** 10.3390/ijms22094337

**Published:** 2021-04-21

**Authors:** Kai Huang, Tao Wu, Ziming Ma, Zhao Li, Haoyuan Chen, Mingxing Zhang, Mingdi Bian, Huijiao Bai, Wenzhu Jiang, Xinglin Du

**Affiliations:** Jilin Province Engineering Laboratory of Plant Genetic Improvement, College of Plant Science, Jilin University, Changchun 130062, China; 15584159829@163.com (K.H.); jidawt@jlu.edu.cn (T.W.); mzm1040722406@163.com (Z.M.); zklizhao@jlu.edu.cn (Z.L.); hychen18@mails.jlu.edu.cn (H.C.); mingxing19@jlu.edu.cn (M.Z.); bianmd@jlu.edu.cn (M.B.); bhj20212021@163.com (H.B.)

**Keywords:** rice, transcription factor, OsWRKY55, drought response, plant growth, *OsAP2-39*

## Abstract

WRKY transcription factors (TFs) have been reported to respond to biotic and abiotic stresses and regulate plant growth and development. However, the molecular mechanisms of WRKY TFs involved in drought stress and regulating plant height in rice remain largely unknown. In this study, we found that transgenic rice lines overexpressing *OsWRKY55* (*OsWRKY55*-OE) exhibited reduced drought resistance. The *OsWRKY55*-OE lines showed faster water loss and greater accumulation of hydrogen peroxide (H_2_O_2_) and superoxide radical (O_2_^−·^) compared to wild-type (WT) plants under drought conditions. *OsWRKY55* was expressed in various tissues and was induced by drought and abscisic acid (ABA) treatments. Through yeast two-hybrid assays, we found that OsWRKY55 interacted with four mitogen-activated protein kinases (MAPKs) that could be induced by drought, including OsMPK7, OsMPK9, OsMPK20-1, and OsMPK20-4. The activation effects of the four OsMPKs on OsWRKY55 transcriptional activity were demonstrated by a GAL4-dependent chimeric transactivation assay in rice protoplasts. Furthermore, OsWRKY55 was able to reduce plant height under normal conditions by decreasing the cell size. In addition, based on a dual luciferase reporter assay, OsWRKY55 was shown to bind to the promoter of *OsAP2-39* through a yeast one-hybrid assay and positively regulate *OsAP2-39* expression. These results suggest that OsWRKY55 plays a critical role in responses to drought stress and the regulation of plant height in rice, further providing valuable information for crop improvement.

## 1. Introduction

As an adverse environmental factor, drought stress greatly constrains plant growth and productivity [1]. To withstand this stress, plants have evolved a complex series of actions that manipulate the expression levels of certain sets of genes. These actions include signal perception and transmission. Transcription factors (TFs) are involved and play a vital role in these signaling cascades [2,3]. In rice, which is often used as a monocot plant model, many TFs have been characterized using forward and/or reverse genetic approaches, including MYB, WRKY, Apetala2 (AP2)/ethylene-responsive factor (ERF), basic region-leucine zipper (bZIP), NAC (NAM, ATAF1/2, CUC1/2), and basic helix-loop-helix (bHLH) [4,5,6,7,8].

Plant-specific WRKY TFs, one of the largest families of transcriptional regulators, are characterized by possessing a WRKY-DNA-binding domain [9]. Investigations of the overexpression or knockdown lines have shown that most WRKY TFs are involved in the plant immune response [10,11,12,13]. WRKY TFs also play crucial roles in various abiotic stress responses. The heterologous overexpression of either *ZmWRKY106* or *ZmWRKY40* in *Arabidopsis* was found to confer drought tolerance [14,15], and the overexpression of *GmWRKY12* in *Arabidopsis* was found to enhance tolerance to drought and salt. Similarly, *GmWRKY54* was found to confer drought tolerance in soybean [16,17], while the overexpression of *OsWRKY76* improved tolerance to cold stress in rice [18]. However, the mechanisms by which WRKY TFs participate in drought stress responses in rice have not been fully elucidated. Plant mitogen-activated protein kinase (MAPK) cascades play important roles in different physiological responses, including biotic and abiotic stresses [19]. A few MAPK cascade genes have been identified to function in response to biotic and abiotic stresses through WRKY TFs [20,21]. OsWRKY30 is phosphorylated by MAP kinases, thereby enhancing drought tolerance in rice [22], and OsBWMK1 mediates defense responses by activating *OsWRKY33* to regulate the expression of several pathogenesis-related genes [23]. OsWRKY53 interacts with OsMPK3/OsMPK6 and inhibits their activity [24].

The roles of several WRKY genes in plant growth and developmental processes, such as seed dormancy, germination, leaf senescence, and plant height, were also identified in previous studies. For example, in rice, the *Oswrky29* mutant displayed enhanced seed dormancy [25]. In *Arabidopsis*, a lack of *WRKY41* reduced primary seed dormancy [26]. The heterologous overexpression of *OsWRKY23* in *Arabidopsis* accelerated leaf senescence in darkness [27]. WRKY53 was found to act as a positive regulator of senescence, while WRKY70 negatively regulated leaf senescence in *Arabidopsis* [28,29]. Moreover, the enhanced expression of *OsWRKY11.2* resulted in a semi-dwarf phenotype [30]. However, the functions of a number of WRKY TFs in rice remain to be elucidated.

Previously, overexpression of the WRKY family gene *OsWRKY55* (LOC_Os03g20550, named Os*WRKY55* by Xie et al. [31] and *OsWRKY31* by Zhang et al. [32]) in rice was shown to enhance resistance to *Magnaporthe grisea* infection and induce the expression of defense-related genes, as well as affect root growth and auxin responses [32]. In this study, we characterized the other underlying roles of *OsWRKY55* in rice. OsWRKY55 not only modulates rice height but is also involved in drought stress. The overexpression of *OsWRKY55* in rice leads to a reduction in drought tolerance and plant height. Our findings reveal that OsWRKY55 plays crucial roles in the responses to drought stress and in the determination of plant height.

## 2. Results

### 2.1. Overexpression of OsWRKY55 Reduces Drought Tolerance in Rice

**Figure 1:**
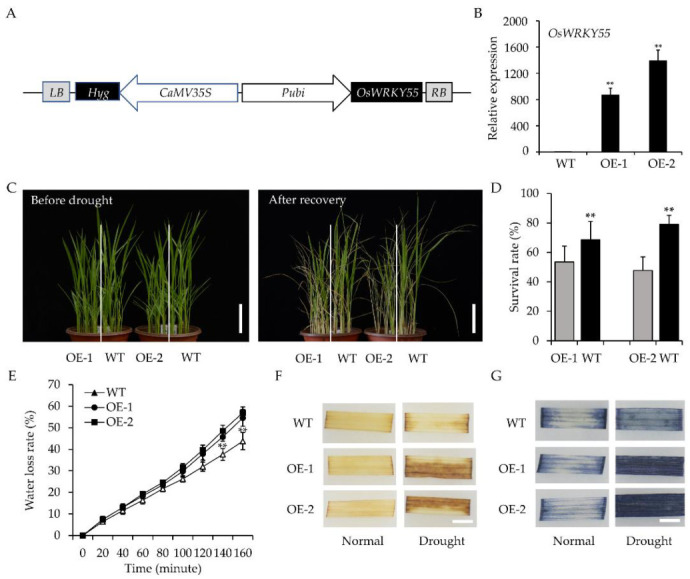
Overexpression of *OsWRKY55* increases the sensitivity of rice seedlings to drought stress. (**A**) Diagrams of the overexpression plasmid of *OsWRKY55*. (**B**) The *OsWRKY55* mRNA level in each genotype was analyzed by quantitative reverse transcription PCR (qRT-PCR). The rice *OsActin* gene was used as the internal control. (**C**) Appearance of wild-type (WT) and transgenic seedlings (OE-1 and OE-2) before drought stress and after 7 days of recovery (rewatering). Scale bars = 5 cm. (**D**) Survival rates of WT and transgenic seedlings after recovery for 7 days. (**E**) Water loss assay of WT, OE-1, and OE-2. (**F**) 3, 3′-diaminobenzidine (DAB) staining showing the level of hydrogen peroxide (H_2_O_2_) in the leaves of WT and transgenic plants under normal and drought-stress conditions. Scale bars = 0.5 cm. (**G**) Nitro-blue tetrazolium (NBT) staining showing the level of superoxide radical (O_2_^−·^) in the leaves of WT and transgenic plants under normal and drought-stress conditions. Scale bars = 0.5 cm. In all graphs, values shown are means ± SD (n = 3), ** *p* < 0.01 (Student’s *t*-test).

Previously, we constructed transgenic rice plants overexpressing different TFs to explore the roles of TFs in response to environmental stresses [33]. We identified two transgenic lines overexpressing *OsWRKY55* by surveying the phenotypes of transgenic lines overexpressing the *WRKY* gene under drought stress treatment. Using quantitative reverse transcription PCR (qRT-PCR), we verified that *OsWRKY55* was significantly up-regulated in two independent transgenic lines, OE-1 and OE-2, and the expression level of the endogenous *OsWRKY55* gene in transgenic rice plants was similar to that in wild type (WT) (Figure 1A,B and Figure S1).

The seedlings overexpressing *OsWRKY55* (*OsWRKY55*-OE) and wild-type (WT) plants were phenotypically indistinguishable under normal conditions. However, leaf wilt was much more severe in the transgenic seedlings than in the WT seedlings under drought stress (Figure 1C). After 7 days of recovery (re-watering), the survival rates of the WT seedlings (≈65–80%) were significantly greater than those of the transgenic *OsWRKY55*-OE seedlings (≈45–53%; Figure 1D), suggesting that *OsWRKY55* negatively regulates drought resistance in rice. This hypothesis was tested by a detached leaf water loss assay. The transgenic *OsWRKY55*-OE lost water faster than the WT (Figure 1E). Since abiotic stress may lead to the accumulation of excessive reactive oxygen species (ROS) in plants, resulting in cell damage, we measured ROS accumulation using histochemical assays. Ultimately, 3, 3′-diaminobenzidine (DAB) staining indicated that under drought conditions, more hydrogen peroxide (H_2_O_2_) was accumulated in the leaves of the *OsWRKY55*-OE plants than in those of the WT plants, as evidenced by the abundance of brown spots in the *OsWRKY55*-OE leaves; there were no visible differences between *OsWRKY55*-OE and WT leaves under normal conditions (Figure 1F). Although both WT and *OsWRKY55*-OE plants showed evidence of superoxide radical (O_2_^−·^) accumulation under drought conditions (as indicated by blue dots on the leaves after nitro-blue tetrazolium (NBT) staining), fewer, lighter dots were also observed on the WT leaves; there were no visible differences between the *OsWRKY55*-OE and WT leaves under normal conditions (Figure 1G). These results indicate that the overexpression of *OsWRKY55* may increase ROS production, thereby decreasing drought resistance.

### 2.2. Expression Pattern of OsWRKY55

**Figure 2:**
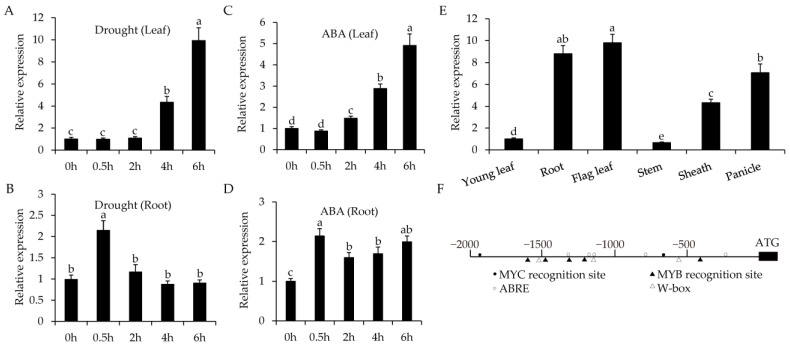
Expression pattern analysis of *OsWRKY55.* (**A**,**B**) Relative expression of *OsWRKY55* in leaves (**A**) and roots (**B**) after drought treatment (dehydration). (**C**,**D**) Relative expression of *OsWRKY55* in leaves (**C**) and roots (**D**) after treatment with 100 µM of the phytohormone abscisic acid (ABA). (**E**) Expression profile of *OsWRKY55* in various rice tissues of the wild-type (WT). (**F**) Distribution of stress-response-related cis-elements in the 2 kb promoter region of *OsWRKY55*. In all graphs, values shown are means ± SD (n = 3). The rice *OsActin* gene was used as the internal control. Different letters above the bars indicate significant differences (*p* < 0.05).

To further explore the function of *OsWRKY55* in rice, we first detected the expression profile of *OsWRKY55* under drought stress and abscisic acid (ABA) treatment. *OsWRKY55* was strongly up-regulated after drought stress. *OsWRKY55* expression peaked in the leaves after 6 h of drought (Figure 2A) and in the roots after 0.5 h of drought (Figure 2B). QRT-PCR analyses showed that the induced expression patterns of *OsWRKY55* in leaves and roots under ABA treatment were similar to those after drought stress (Figure 2C,D). In addition, we also assessed the tempo-spatial expression profile of this gene via qRT-PCR. *OsWRKY55* was constitutively expressed in all tissues analyzed, with relatively higher expression levels in roots and flag leaves and relatively lower expression levels in young leaves and stems (Figure 2E). As the preliminary results suggested that *OsWRKY55* is associated with drought response, we next analyzed the promoter sequence of the *OsWRKY55* gene using the PlantCARE database (http://sphinx.rug.ac.be:8080/PlantCARE/, accessed on 15 September, 2020). Analysis of the *OsWRKY55* gene promoter sequence identified many putative stress response-related cis-elements, including the MYB recognition site (5 hits), ABA-responsive element (ABRE) (5 hits), W-box (3 hits), and MYC recognition site (2 hits) (Figure 2F).

### 2.3. OsWRKY55 Interacts with Several OsMPKs

**Figure 3:**
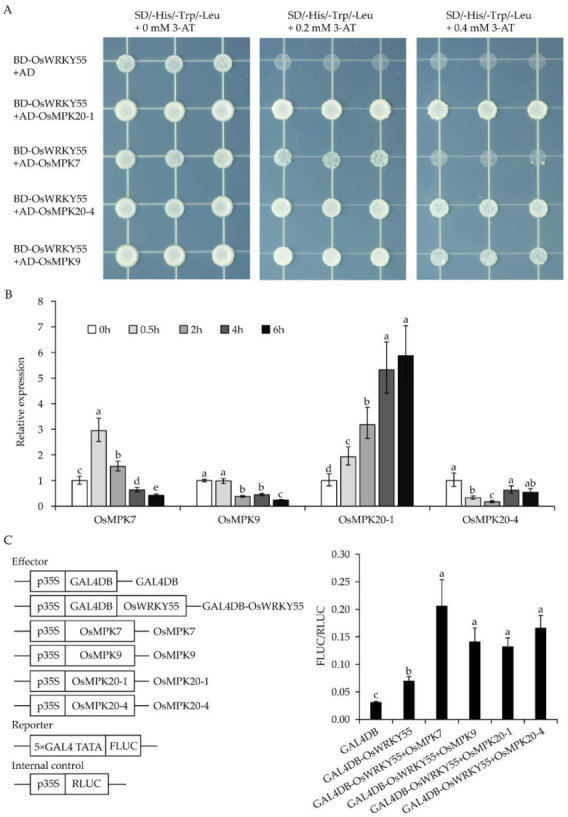
OsMPKs interact with OsWRKY55 and increase its transcription activity. (**A**) The interactions between OsWRKY55 and four OsMPKs in the yeast two-hybrid assays. Growth phenotypes of yeast cells on selective media plates (SD/-Trp-Leu-His) containing 0, 0.2, or 0.4 mM 3-amino-1, 2, 4-triazole (3-AT). (**B**) Relative expressions of *OsMPK7*, *OsMPK9*, *OsMPK20-1*, and *OsMPK20-4* after drought treatment (dehydration). (**C**) GAL4-dependent chimeric transactivation assay of OsWRKY55 in rice protoplasts. A schematic illustration of the effector and reporter vectors is shown on the left. FLUC, firefly luciferase; RLUC, renilla luciferase. The right part is the transcription activities by co-transformation of different effector vector(s) with the reporter vector and internal control. Values shown are means ± SD (n = 3). The rice *OsActin* gene was used as the internal control. Different letters above the bars indicate significant differences (*p* < 0.05).

To determine whether OsWRKY55 interacts with OsMPKs, the full-length coding sequences (CDSs) of 15 *OsMPKs* [34] were fused to separate pGADT7 as prey, and pGBKT7-OsWRKY55 was used as bait in the yeast two-hybrid assays. The results showed that all transformants grew well on the SD/-Trp-Leu-His plates containing 0 mM 3-amino-1, 2, 4-triazole (3-AT) (Figure 3A and Figure S2) due to the auto-transcriptional activity of OsWRKY55 in yeast [32]. Only three transformants (carrying BD-OsWRKY55/AD-OsMPK20-1, BD-OsWRKY55/AD-OsMPK20-4, or BD-OsWRKY55/AD-OsMPK9) grew well on the SD/-Trp-Leu-His plates containing 0.2 and 0.4 mM 3-AT (Figure 3A and Figure S2). However, the growth of cells carrying BD-OsWRKY55/AD-OsMPK7 was reduced on the 0.2 mM 3-AT plate and inhibited on the 0.4 mM 3-AT plate (Figure 3A). Negative control yeast cells carrying BD-OsWRKY55/AD did not grow well on SD/-Trp-Leu-His plates containing 0.2 and 0.4 mM 3-AT. These results indicate that, in yeast, OsWRKY55 interacts strongly with OsMPK20-1, OsMPK20-4, and OsMPK9 but weakly with OsMPK7 (Figure 3A).

To examine the possible involvement of these four *OsMPKs* in the drought stress response, we assessed the expression patterns of *OsMPKs* under drought stress via qRT-PCR. We found that the expression level of *OsMPK7* was increased within 0.5 h and that *OsMPK9* expression was down-regulated rapidly after 2 h of drought treatment. Under drought stress, the expression of *OsMPK20-1* was significantly induced, and *OsMPK20-4* was suppressed after 0.5 h, but the expression resumed slightly after 4 h (Figure 3B).

To further explore whether the four OsMPKs affect the activity of OsWRKY55, a GAL4-dependent chimeric transactivation assay in rice protoplasts was performed. The results illustrated the activation effects of the four OsMPKs on OsWRKY55 transcriptional activity (Figure 3C), which also suggested that the interactions between OsWRKY55 and OsMPKs occurred in vivo.

### 2.4. Overexpression of OsWRKY55 Reduces Plant Height

**Figure 4:**
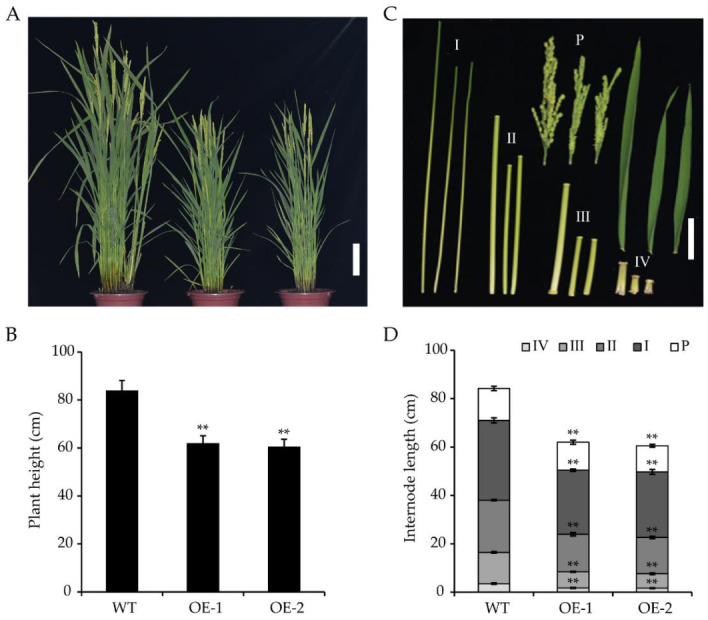
Overexpression of *OsWRKY55* reduces plant height. (**A**) Phenotypes of wild-type (WT) plants and transgenic lines overexpressing *OsWRKY55* (OE-1 and OE-2). Scale bar = 10 cm. (**B**) Heights of mature WT and OE plants. (**C**) Panicles (P) and internodes (I-IV) of mature WT and *OsWRKY55*-OE plants. Scale bar = 5 cm. (**D**) Lengths of panicles (P) and internodes (I-IV) in mature WT and *OsWRKY55*-OE plants. In all graphs, values shown are means ± SD (n ≥ 15), ** *p* < 0.01 (Student’s *t*-test).

**Figure 5:**
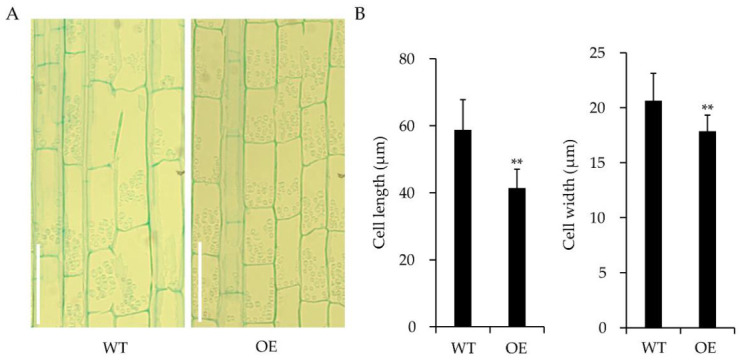
Overexpression of *OsWRKY55* decreases cell size. (**A**) Longitudinal sections of the uppermost internodes of the main culms of the wild-type (WT) plants and transgenic lines overexpressing *OsWRKY55* (*OsWRKY55*-OE-1). Scale bars = 50 μm. (**B**) Cell size comparison of the uppermost internodes of the main culms in WT and *OsWRKY55*-OE (OE-1) lines. Values shown are means ± SD (n = 3), ** *p* < 0.01 (Student’s *t*-test).

OsWRKY55 was not only associated with the drought stress response but was also observed to affect plant growth. The transgenic *OsWRKY55*-OE lines grew more slowly than the WT plants (Figure 4A). At maturity, the two *OsWRKY55*-OE lines were significantly shorter than those of the WT plants—OE1 and OE2 were 25.4% and 27.8% shorter than the WT, respectively (Figure 4B). All four internodes of each *OsWRKY55*-OE line were significantly shorter than the corresponding WT internodes (Figure 4C,D). A microscopic examination of the longitudinal sections of the uppermost internodes of the main culms of the WT and *OsWRKY55*-OE (OE-1) plants showed that there were noticeable differences in cell shape between the WT and transgenic plants. Indeed, there were significant differences in both cell length and cell width between the WT and *OsWRKY55*-OE (OE-1) plants (Figure 5A,B). Thus, our results indicate that *OsWRKY55* affects plant height by negatively regulating cell expansion.

### 2.5. OsAP2-39 Is Directly Regulated by OsWRKY55

**Figure 6:**
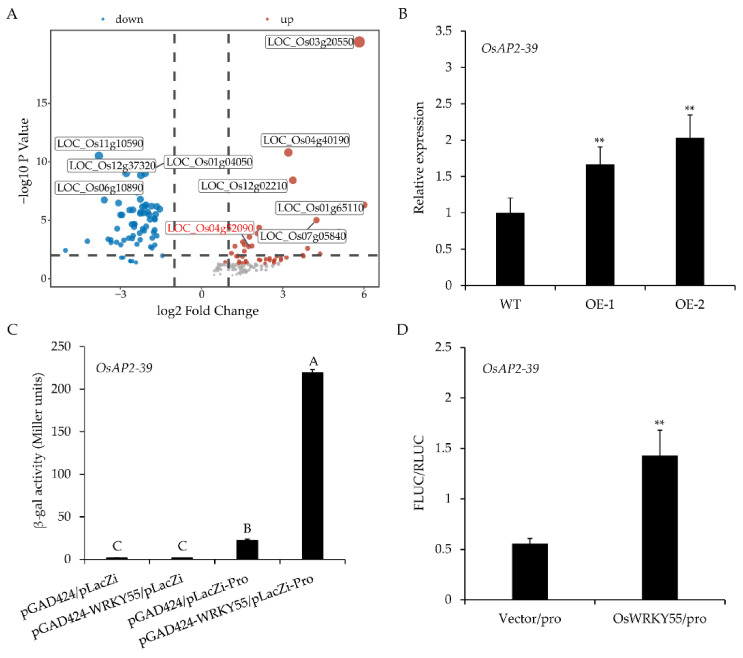
OsWRKY55 positively regulates the expression of *OsAP2-39*. (**A**) Identification of differentially expressed genes (DEGs) in transgenic lines overexpressing *OsWRKY55* compared to the wild type (WT) under normal conditions via RNA-sequencing (RNA-seq) analysis. The blue and red spots represent down-regulated and up-regulated genes, respectively. The sizes of the spots indicate the -log_10_FDR. (**B**) Relative expression of the *OsAP2-39* gene in WT plants and transgenic lines overexpressing *OsWRKY55* (OE-1 and OE-2). The rice *OsActin* gene was used as the internal control. Values shown are means ± SD (n = 3), ** *p* < 0.01 (Student’s *t*-test). (**C**) β-galactosidase activity levels in co-transformed yeast cells. Values shown are means ± SD (n = 3). Different uppercase letters above the bars indicate significant differences (least significant differences, *p* < 0.01). (**D**) Transcriptional activity of *OsAP2-39* in rice protoplasts harboring the effector vector pAN580-OsWRKY55 compared to that in protoplasts carrying the empty vector. The transient expression of FLUC driven by the promoter of *OsAP2-39* was normalized to the internal control reporter (RLUC). Values shown are means ± SD (n = 3), ** *p* < 0.01 (Student’s *t*-test).

To elucidate the molecular function of *OsWRKY55*, we identified the target genes of OsWRKY55 through the genome-wide expression profile changes in WT and *OsWRKY55*-OE lines via an RNA-sequencing (RNA-seq) assay under normal growth conditions at the four-leaf stage. The result revealed that about 162 genes in overexpressing *OsWRKY55* rice experienced two-fold changes compared to the genes in the WT rice. Among these genes, 98 were up-regulated in the OE lines. Although the predicted functions of the up-regulated genes were extremely diverse, there were many stress-related genes, such as *OsAP2-39* (LOC_Os04g52090), *OsERF48*/*OsDRAP1* (LOC_Os08g31580), *OsFbx352* (LOC_Os10g03850), *OsRAV2* (LOC_Os01g04800), *OsMYB48* (LOC_Os01g74410), and Zinc finger protein (LOC_Os03g55540, LOC_Os03g60570, LOC_Os01g61420, LOC_Os02g45710) (Table S2). Therefore, these genes might be direct or indirect targets of *OsWRKY55*. As *OsAP2-39*, the APETALA-2-like transcription factor is known to negatively regulate plant growth and drought tolerance [35], we further investigated an experimentally verified gene, *OsAP2-39* (Figure 6A). The up-regulation of *OsAP2-39* in transgenic lines overexpressing *OsWRKY55* was verified by qRT-PCR (Figure 6B).

To further identify whether *OsAP2-39* is the target gene of OsWRKY55, a yeast one-hybrid assay and a dual luciferase reporter assay were performed. The β-galactosidase activity level in the yeast cells co-transformed with pLacZi-Pro and pGAD424-OsWRKY55 was significantly greater than that of the control (a ≈10-fold increase in activity; Figure 6C), indicating that OsWRKY55 binds to the *OsAP2-39* promoter in yeast. Furthermore, the dual luciferase reporter assay showed that the FLUC/RLUC ratio, which reflects transcriptional activity, was significantly up-regulated in rice protoplasts harboring the effector vector pAN580-OsWRKY55 compared to that in protoplasts carrying the vector pAN580 (Figure 6D). These results suggest that *OsAP2-39* is a target gene of OsWRKY55.

## 3. Discussion

Rice is negatively affected by various abiotic and biotic stresses, resulting in serious yield losses worldwide. Previous studies have identified that many WRKY TFs participate in the rice response to biotic stresses [36]. However, to date, our understanding of the molecular mechanisms by which WRKY TFs in rice are involved in abiotic stresses, especially drought stress, remains largely unknown. Furthermore, the roles of WRKY TFs in the regulation of growth and development in rice have not yet been studied extensively. A prior study showed that *OsWRKY55* enhanced resistance against infection with *M. grisea* and affected root growth and auxin response [32]. In the present work, we focused on the response of *OsWRKY55* to drought stress and the regulation of plant height. Under drought treatment, by withholding the water supply, *OsWRKY55*-OE plants became more sensitive to drought stress than WT plants (Figure 1C,D), which is likely because the *OsWRKY55*-OE plants lost water at a faster rate (Figure 1E) and had fewer, shorter lateral roots than the WT plants [32]. Furthermore, the *OsWRKY55*-OE plants accumulated more H_2_O_2_ and O_2_^−^ under drought conditions (Figure 1F,G). The associated increase in oxidative damage might also explain the increased susceptibility of the *OsWRKY55*-OE transgenic lines to drought. We also found that the overexpression of *OsWRKY55* reduced plant growth by decreasing cell size under normal conditions: *OsWRKY55*-OE plants were shorter than WT plants, with smaller, thinner cells in the internodes (Figure 4 and Figure 5). A previous study revealed that *OsWRKY55* is an auxin-inducible gene and that the overexpression of *OsWRKY55* enhances auxin-related phenotypes in rice [32]. Auxin regulates a variety of processes during plant development [37]. However, it remains unclear whether the involvement of *OsWRKY55* in auxin signaling leads to the dwarfism observed in the *OsWRKY55*-OE plants.

MAPK cascades in plants are involved in various physiological functions. WRKY TFs and MAPK cascades have been shown to modulate plant stress response [19,38,39,40,41,42]. Under biotic and abiotic stress, many WRKY TFs are activated by MAPKs. For example, OsMPK6 phosphorylates and activates OsWRKY45. However, under cold and salinity stress, a tyrosine protein phosphatase dephosphorylates and inactivates OsMPK6, which reduces OsWRKY45 defense [43]. OsMPK7and OsMPK20-4 interact with OsWRKY30, and OsMPK7 further phosphorylates OsWRKY30, which are both crucial processes for OsWRKY30 to confer drought tolerance in rice [22]. In this study, we demonstrated that OsWRKY55 interacts with four different MAPKs in yeast, including OsMPK7, OsMPK9, OsMPK20-1, and OsMPK20-4 (Figure 3A), and that the four OsMPKs can activate the transcriptional activity of OsWRKY55. The qRT-PCR results showed that these four *OsMPKs* may be related to drought stress response (Figure 3B). These results suggest that the decreased drought tolerance associated with *OsWRKY55* may, to a large extent, be due to the interactions between OsWRKY55 and these four OsMPKs. OsWRKY30 and OsWRKY55 can be activated by OsMPK7, but they confer different drought tolerance levels in rice, which indicates that the regulatory network of responses to drought stress in rice is complex. Overexpression of the *OsWRKY55* gene was found to enhance resistance against infection with *M. grisea* [32]. It was suggested that OsWRKY55 plays an opposite role in biotic and abiotic stress, which may be due to the regulation of different downstream genes by OsWRKY55.

AP2/ERF proteins play important roles in plant responses to biotic and abiotic stress, as well as in the growth and development in rice. For example, *OsAP2-39*, a member of the APETALA2 (AP2) family, affects dehydration tolerance and plant height in rice by controlling the ABA/gibberellin (GA) balance [35]. However, overexpressing *OsAP2-39* lines display different response results depending on the different ways in which they are grown under drought stress. When grown in the same pot, transgenic *OsAP2-39* plants exhibited lower dehydration tolerance than WT plants, which is likely due to smaller root systems of overexpressing *OsAP2-39* lines, which are similar to those of the drought-sensitive phenotype of *OsWRKY55*-OE plants. When grown in separate pots, the responses were reversed, which was likely because the transpiration rates of the WT plants were greater. The definitive conclusion, that overexpressing *OsAP2-39* lines had lower dehydration tolerance than the WT, was reached via an excised leaf water loss assay. It was reported that an ERF protein, OsDERF1, activates *OsERF3* and *OsAP2-39* to negatively modulate ethylene synthesis and drought tolerance in rice [44]. The present study revealed that OsWRKY55 can bind to the promoter of *OsAP2-39* to up-regulate the expression of *OsAP2-39* (Figure 6), suggesting that the OsWRKY55 and OsAP2-39 transcriptional cascade negatively modulates drought response. OsDERF1 and OsWRKY55 both regulate drought tolerance by activating *OsAP2-39* in rice, but whether they function in the same pathway remains to be elucidated.

In conclusion, the overexpression of *OsWRKY55* could reduce drought resistance and plant height in rice. Four OsMPKs interacted with OsWRKY55 and activated OsWRKY55. Moreover, OsWRKY55 was able to directly regulate *OsAP2-39*. This work demonstrates the novel function of *OsWRKY55* in rice.

## 4. Materials and Methods

### 4.1. Plant Materials and Growth Conditions

Wild-type rice seedlings (WT, *Oryza sativa japonica* cv Kitaake) and two homozygous T3 transgenic rice lines overexpressing *OsWRKY55* (OE-1 and OE-2) were used in these experiments. To generate *OsWRKY55*-OE overexpression lines, the full-length coding sequence (CDS) of *OsWRKY55* was amplified from rice seedlings of Kitaake and cloned into the vector pCUbi1390 under the control of the maize ubiquitin (Ubi) promoter. The constructed vector *pUbi:OsWRKY55* was introduced into rice (Kitaake) using *Agrobacterium tumefaciens* (EHA105)-mediated transformation. The homozygous lines were selected via hygromycin resistance evaluation. Rice overexpressing *OsWRKY55* is denoted as OE, and the different transgenic lines are indicated with numbers.

Seeds were submerged in water at 28 °C for 3 days. Then, the uniformly germinated seeds were transplanted into containers filled with Yoshida’s culture solution and boxes filled with soil from a paddy field in a growth chamber (28 °C, 80% relative humidity and a 14/10 h day/night photoperiod), respectively.

### 4.2. Stress Treatments

Phenotypic analysis of drought stress was conducted at the seedling stage. When *OsWRKY55*-OE lines and WT plants growing in the soil reached the four-leaf stage, irrigation was withheld for 7 days, and then the seedlings were allowed to recover for 7 days. Seedlings that did not grow were considered not to have survived. The survival rates were calculated as the percentage of seedlings that survived among the total treated seedlings. WT and transgenic plants were grown to maturity in the paddy field under normal conditions. At the booting stage, we measured lengths of the panicles and internodes and plant heights.

To determine the *OsWRKY55* gene expression profile under stress conditions, four-leaf-stage WT rice seedlings growing in the hydroponic culture medium were subjected to drought stress (by removing the water supply) and phytohormone ABA treatment (100 µM ABA added to the culture medium) before being sampled at 0, 0.5, 2, 4, and 6 h. To characterize the spatiotemporal expression of *OsWRKY55*, young leaves and roots were collected from the WT seedlings at the four-leaf stage in the soil under normal conditions, and samples of the flag leaves, stems, sheaths, and panicles were collected from WT plants at the booting stage described above.

### 4.3. Water Loss Assay

The *OsWRKY55*-OE and WT plants were grown in pots under normal growth conditions for 4 weeks. The leaves were detached from the plants and weighed immediately as the initial weight. The samples were exposed to air at room temperature and then weighed at the same time intervals (20 min) after being cut down. The rate of water loss was calculated based on the initial weights of the samples. Three replicates were made for each line.

### 4.4. Quantification of Gene Expression

Total RNA was extracted from each sample using the RNAprep Pure Plant Kit (Tiangen Biotech). First-strand cDNA was synthesized with 2 μg of total RNA using a RevertAid First Strand cDNA Synthesis Kit (Thermo Scientific Fermentas). Quantitative RT-PCR reaction was performed using a Mx3005P instrument (Stratagene) with the SYBR Green Real-time PCR Master Mix (Toyobo) according to the following conditions: 1 cycle of 2 min at 95 °C, 40 cycles of 15 s at 95 °C, and 30 s at 60 °C, followed by 1 cycle of 60 s at 95 °C, 30 s at 55 °C, and 30 s at 95 °C for the melting curve analysis. The rice *OsActin* gene and *Ubiquitin5* gene (Supplemental Figure S3) were used as the internal controls against which to calculate relative expression. Relative expression levels were calculated using the 2^−ΔΔCt^ method [45]. The experiment was conducted using three biological replicates. The primers used are listed in Table S1.

### 4.5. RNA Sequencing

Four-leaf-stage WT and the *OsWRKY55*-OE plants under normal conditions were sampled for RNA sequencing (RNA-seq). Total RNA of the leaves was extracted using the RNAprep Pure Plant Kit (Tiangen Biotech). cDNA library construction and next-generation sequencing were performed by Biomarker Bio-Tech Company (Beijing, China) on an Illumina Hiseq2500 platform (2 × 101 bp). Three biological replicates were performed. The raw reads were filtered using the FASTQ_Quality_Filter tool from the FASTX-Toolkit (http://hannonlab.cshl.edu/fastx_toolkit, accessed on 11 May, 2020). Next, the clean reads were mapped to the rice reference genome (MSU, Rice Genome Annotation Release 7) using Hisat2, a spliced read mapper for RNA-seq (http://ccb.jhu.edu/software/hisat2/index.shtml, accessed on 11 May, 2020). Gene expression profiles were analyzed using Stringtie (Transcript assembly for RNA-seq), and transcript abundance was measured using the Fragment Per Kilobase of transcript sequence per Millions base pairs Sequenced (FPKM) method. Differentially expressed genes (DEGs) were identified using the DESeq package in R. The volcano map of DEGs was made using R package ggplot2.

### 4.6. Yeast Two-Hybrid Assay

To identify the interactions of OsWRKY55 and OsMPKs in yeast, a yeast two-hybrid assay was carried out according to the manufacturer’s instructions (Clontech). The full-length coding DNA sequence (CDS) of *OsWRKY55* was fused to the pGBKT7 vector (BD-OsWRKY55) as bait, and the full-length CDSs of the *OsMPKs* were cloned into separate pGADT7 vectors as prey. The bait vector was co-transformed with each prey vector into the Y2H Gold yeast strain and cultured on SD/-Trp-Leu plates at 30 °C for 2–3 days. Positive transformants were then cultivated on SD/-Trp-Leu-His plates containing 0, 0.2, or 0.4 mM 3-amino-1, 2, 4-triazole (3-AT) at 30 °C for 3 days.

### 4.7. Yeast One-Hybrid Assay

For the yeast one-hybrid assay, the promoter (≈1500 bp) of *OsAP2-39* was cloned into the reporter vector pLacZi (pLacZi-Pro). The full-length CDS of *OsWRKY55* was amplified and fused to the vector pGAD424 (Clontech), which encodes the activation domain of the yeast GAL4 transcriptional activator. Then, the reporter vector pLacZi-Pro was digested using a restriction enzyme (either *NcoI* or *ApaI*) and co-transformed with the fusion protein into the yeast strain YM4271 (Clontech). The transformants were cultured on SD/-Leu-Ura plates at 30 °C for 3 days. Putative positive clones were identified using DNA sequencing. Finally, β-galactosidase liquid assays were performed to quantify the DNA–protein interactions, following the instructions of the manufacturer (Clontech).

### 4.8. GAL4-Dependent Chimeric Transactivation Assay

The assay was performed in rice protoplasts, as described previously [46]. The *OsWRKY55* coding region was cloned into the effector vector with the GAL4 DNA-binding domain, while the full-length coding regions of OsMPK7, OsMPK9, OsMPK20-1, and OsMPK20-4 were cloned into the effector plasmid without the GAL4 DNA-binding domain. GAL4DB was used as a negative effector. The reporter was a plasmid containing the firefly luciferase (FLUC) gene. A plasmid harboring the Renilla LUC (RLUC) gene under control of the *CaMV* 35S promoter was used as an internal control. The effector plasmids were co-transformed with the reporter plasmid and internal control into rice protoplasts. After incubation at 28 °C for 12–16 h, the protoplasts were collected, and the results were reported as the ratios between the activity of FLUC and RLUC using a luciferase activity assay (Promega).

### 4.9. Dual Luciferase Reporter Assay

For the dual luciferase reporter assay, the *OsAP2-39* promoter (~1500 bp) was inserted into the vector pGreen II 0800 (which contained a firefly luciferase (FLUC) reporter gene and an internal control Renilla luciferase (RLUC) driven by the *CaMV* 35S promoter) to generate the reporter vector. The CDS of *OsWRKY55* was inserted into the pAN580 vector to generate the effector vector. Then, the reporter vector was co-transformed into rice protoplasts via PEG-mediated transformation [47] with either the empty pAN580 vector (as the negative control) or the effector vector (pAN580-OsWRKY55). After 12–16 h of culture at 28 °C, the FLUC and RLUC activity levels in the rice protoplasts were measured using a Dual-Luciferase Reporter Assay System (E1910, Promega). The transient expression of LUC, driven by the promoter of *OsAP2-39*, was normalized to the expression of the internal control reporter (RLUC).

### 4.10. Histochemical Analysis

For 3, 3′-diaminobenzidine (DAB) staining, rice leaves were immersed in 1 mg mL^–1^ DAB in 50 mM Tris-acetate buffer (pH 5.0) for 12 h at room temperature in the dark. To detect superoxide radical (O_2_^−·^), leaves were incubated in 1 mg mL^–1^ nitro-blue tetrazolium (NBT) in a 10 mM potassium phosphate solution (pH 7.8) at 25 °C for 12 h in the dark. Then, the leaves were de-stained with 95% ethanol for 10 min at 100 °C. De-staining was repeated several times until the chlorophyll was completely removed.

WT and *OsWRKY55*-OE (OE-1) internodes were immobilized in paraffin and sectioned as described previously [48]. In brief, the uppermost internodes of the main culms of the WT and *OsWRKY55*-OE (OE-1) plants were longitudinally sectioned, fixed in formaldehyde/glacial acetic acid/70% ethanol (1:1:18, *v*/*v*/*v*) for 48 h, and softened with 15% hydrofluoric acid for 2 weeks. Then, the samples were dehydrated using a graded ethanol series. The dehydrated samples were embedded in paraffin (Sigma-Aldrich) and micro-sectioned to a thickness of 8 µm using a microtome. The sections were stained with toluidine blue and viewed under a light microscope after paraffin removal with xylene.

### 4.11. Accession Numbers

Sequence data from this article can be found in the MSU Rice Genome Annotation Project (http://rice.plantbiology.msu.edu/analyses_search_locus.shtml, accessed on 18 April, 2020) databases under the following accession numbers: *OsWRKY55/OsWRKY31* (LOC_Os03g20550), *OsMPK7* (LOC_Os05g49140), *OsMPK9* (LOC_Os05g50560), *OsMPK20-1* (LOC_Os01g43910), *OsMPK20-4* (LOC_Os01g47530), *OsMPK3* (LOC_Os03g17700), *OsMPK4-1* (LOC_Os10g38950), *OsMPK6* (LOC_Os06g06090), *OsMPK14* (LOC_Os02g05480), *OsMPK16* (LOC_Os11g17080), *OsMPK17-1* (LOC_Os06g49430), *OsMPK17-2* (LOC_Os02g04230), *OsMPK20-3* (LOC_Os06g26340), *OsMPK4-2* (LOC_Os06g48590), *OsMPK21-1* (LOC_Os05g50120), *OsMPK21-2* (LOC_Os01g45620), *OsActin* (LOC_Os03g50885), *Ubiquitin5* (LOC_Os01g22490), and *OsAP2-39* (LOC_Os04g52090).

## Supplementary Materials

The following are available online at https://www.mdpi.com/article/10.3390/ijms22094337/s1.
