# Supplementary material for: Rice Transcription Factor OsWRKY55 Is Involved in the Drought Response and Regulation of Plant Growth
Source: Int J Mol Sci. 2021 Apr 21;22(9):4337. doi: 10.3390/ijms22094337 (PMC8122443; doi:10.3390/ijms22094337)
Supplement: Supplementary file 1 [file ijms-22-04337-s001.zip › Supplementary Files.pdf]

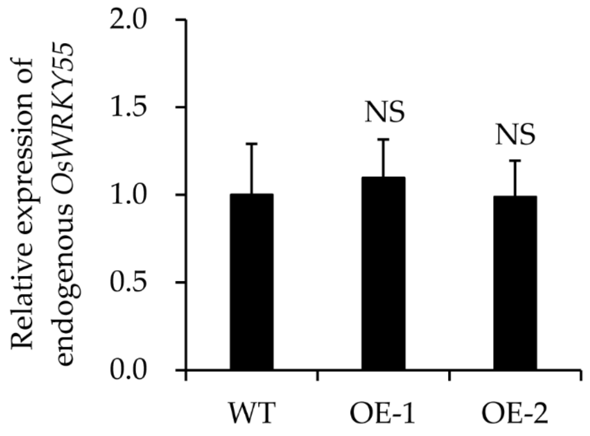


**Figure S1.** Endogenous *OsWRKY55* mRNA level in each genotype was analyzed by quantitative reverse transcription PCR (qRT-PCR). The rice *OsActin* gene was used as the internal control. Values shown are means ± SD (n = 3), Student's *t*-test. WT, wild-type; OE, *OsWRKY55*-overexpressing line; NS, not significant.

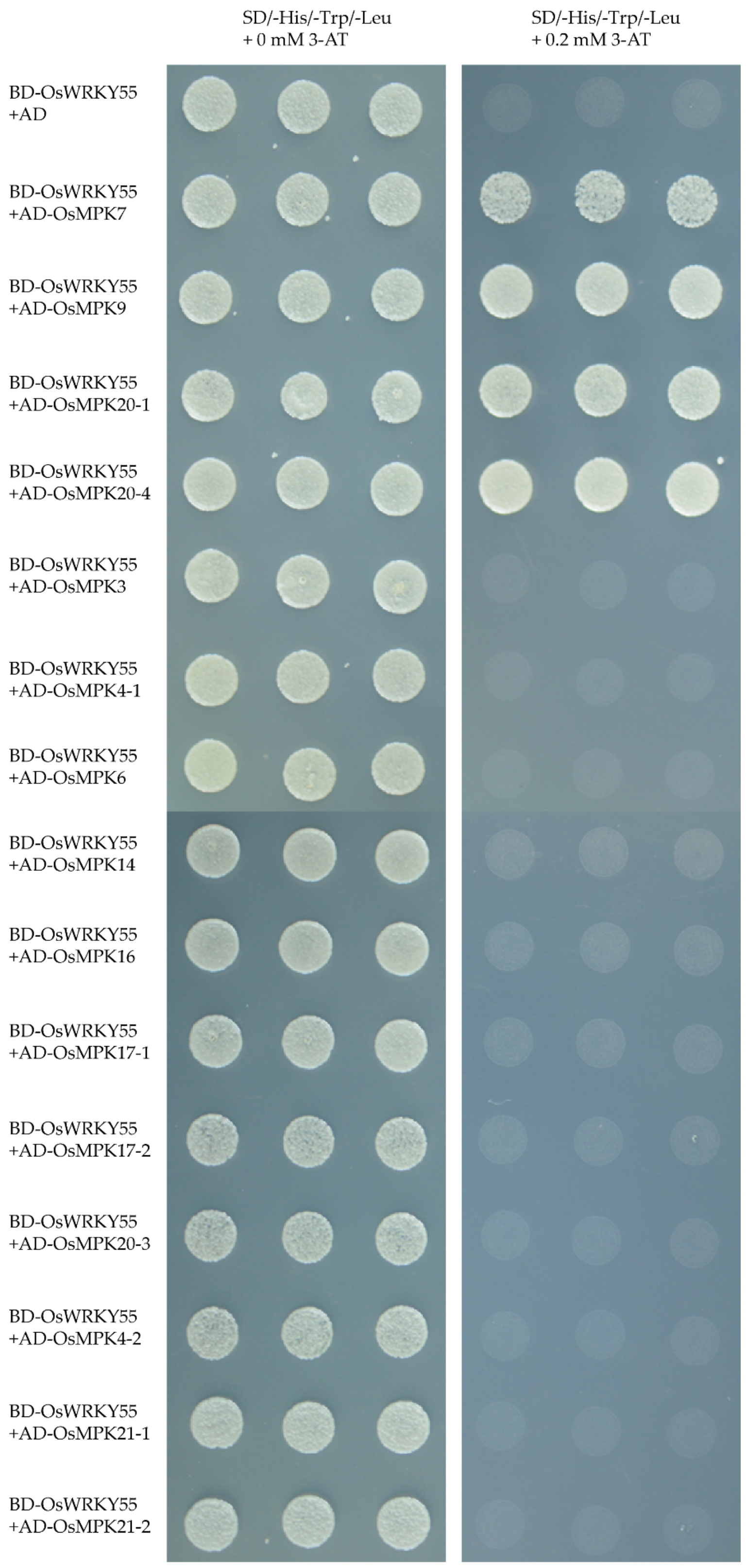


**Figure S2.** The interactions between OsWRKY55 and 15 OsMPKs in yeast two-hybrid assays. Growth phenotypes of yeast cells on selective media plates (SD/-Trp-Leu-His) containing 0 or 0.2 mM 3-amino-1, 2, 4-triazole (3-AT).

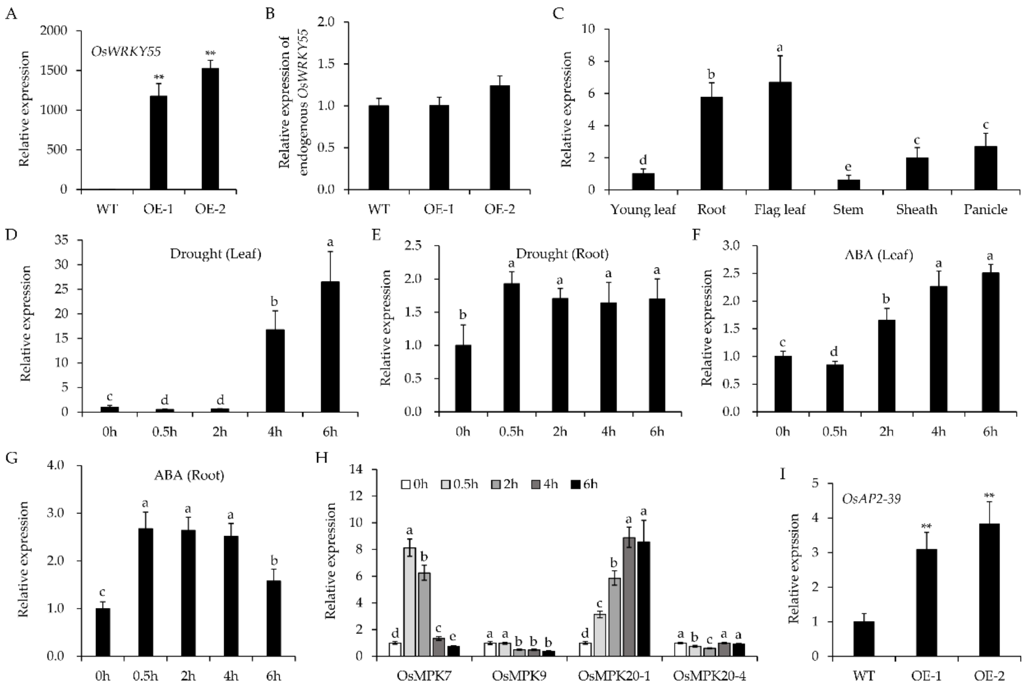


**Figure S3.** All quantitative reverse transcription PCR (qRT-PCR) in this study used the gene *Ubiquitin5* as the inner control. **(A)** The *OsWRKY55* mRNA level in each genotype was analyzed by qRT-PCR. **(B)** Endogenous *OsWRKY55* mRNA level in each genotype was analyzed. **(C)** Expression profile of *OsWRKY55* in various rice tissues of the wild-type (WT). **(D and E)** Relative expression of *OsWRKY55* in leaves (D) and roots (E) after drought treatment (dehydration). **(F and G)** Relative expression of *OsWRKY55* in leaves (F) and roots (G) after treatment with 100 μM of the phytohormone abscisic acid (ABA). **(H)** Relative expressions of *OsMPK7*, *OsMPK9*, *OsMPK20-1*, and *OsMPK20-4* after drought treatment (dehydration). **(I)** Relative expression of the *OsAP2-39* gene in WT plants and transgenic lines overexpressing *OsWRKY55* (OE-1 and OE-2). Values shown are means ± SD (n = 3), ** $P < 0.01$ (Student's *t*-test). Different letters above the bars indicate significant differences ($P < 0.05$).

**Table S1.** Primers used in this study.

| Gene | Primer sequence | Experiments |
|---|---|---|
| *OsWRKY55* | F: GCCGAGTCCGCATCAATC | qRT-PCR |
| | R: TTCTCGCCGTACTTCCTCCA | qRT-PCR |
| | F: AGGAGGACCTGCATATGATGTCTCCTGTGCCGAGTCC | Cloning |
| | R: GCAGGTCGACGGATCCTTACAAAAAATGGAAGAATC | Cloning |
| | F: AAAAGAGATCGAATTCATGTCTCCTGTGCCGAGTCC | Cloning |
| | R: GCAGGTCGACGGATCCTTACAAAAAATGGAAGAATC | Cloning |
| | F: CGGAGCTAGCTCTAGAATGTCTCCTGTGCCGAGTCC | Cloning |
| | R: TGCTCACCATGGATCCCAAAAAATGGAAGAATCTGA | Cloning |
| *OsAP2-39* | F: GATCTGAGCCTCGGGATGTCGG | qRT-PCR |
| | R: AGGCGAGCTACCTCCTTCTTGACG | qRT-PCR |
| | F: CGGTATCGATAAGCTTTTGTTGCGACAGACCAACACTAG | Cloning |
| | R: TAGAACTAGTGGATCCGTCCGTTCTTGTTCGGGTCG | Cloning |
| | F: TGAAAAGCTTGAATTCTTGTTGCGACAGACCAACACTAG | Cloning |
| | R: GAGCACATGCCTCGAGGTCCGTTCTTGTTCGGGTCG | Cloning |
| *OsActin* | F: CATTGGTGCTGAGCGTTTCC | qRT-PCR |
| | R: CTCCTTGCTCATCCTGTCAGC | qRT-PCR |
| *Ubiquitin5* | F: ACCACTTCGACCGCCACTACT | qRT-PCR |
| | R: ACGCCTAAGCCTGCTGGTT | qRT-PCR |
| *OsMPK3* | F: GGAGGCCAGTGAATTCATGGACGGGGCGCCGGTG | Cloning |
| | R: CGAGCTCGATGGATCCCTAGTACCGGATGTTTGG | Cloning |

| | | |
|---|---|---|
| *OsMPK4-1* | F: GGAGGCCAGTGAATTCATGGATTCCTCCTCCGGC | Cloning |
| | R: CGAGCTCGATGGATCCTTAGTAGGGAGGATCCGG | Cloning |
| *OsMPK6* | F: GGAGGCCAGTGAATTCATGGACGCCGGGGCGCAG | Cloning |
| | R: CGAGCTCGATGGATCCCTACTGGTAATCAGGGTT | Cloning |
| *OsMPK14* | F: GGAGGCCAGTGAATTCATGGCGATCATGGTGGAT | Cloning |
| | R: CGAGCTCGATGGATCCTCATCGGGCACTCATTGC | Cloning |
| *OsMPK16* | F: GGAGGCCAGTGAATTCATGGACTTCTTTACCGAG | Cloning |
| | R: CGAGCTCGATGGATCCTCAGGGGTAAGAGCCTCG | Cloning |
| *OsMPK17-1* | F: GGAGGCCAGTGAATTCATGGGGGGAGGGGGCACG | Cloning |
| | R: CGAGCTCGATGGATCCCTAGGAGTGCATCCTGGA | Cloning |
| *OsMPK17-2* | F: GGAGGCCAGTGAATTCATGGAGTTCTTTACGGAA | Cloning |
| | R: CGAGCTCGATGGATCCCTAGGAGAGCATCCTGGT | Cloning |
| *OsMPK20-3* | F: GGAGGCCAGTGAATTCATGCAGACCAGCAATTTT | Cloning |
| | R: CGAGCTCGATGGATCCCTAGGTGATTCTATACGT | Cloning |
| *OsMPK4-2* | F: GGAGGCCAGTGAATTCATGGCGATGATGGTGGAC | Cloning |
| | R: CGAGCTCGATGGATCCTCACATATTCACTCCTGC | Cloning |
| *OsMPK21-1* | F: GGAGGCCAGTGAATTCATGGGCGGCCGCGCCCCGC | Cloning |
| | R: CGAGCTCGATGGATCCTCAGGTTTTCAGTTGAGC | Cloning |
| *OsMPK21-2* | F: GGAGGCCAGTGAATTCATGGATGCTAAGAAGGGC | Cloning |
| | R: CGAGCTCGATGGATCCTTATGTTTTCAATTGAGC | Cloning |
| *OsMPK7* | F: GGAGGCCAGTGAATTCATGCCTGAGGCAAATGCG | Cloning |
| | R: CGAGCTCGATGGATCCCTAGTACATCCTTGAAAC | Cloning |

| | | |
|---|---|---|
| *OsMPK9* | F: GGAGGCCAGTGAATTCATGAGAAAGAAAGATCCAGT | Cloning |
| | R: CGAGCTCGATGGATCCCTAATACATCCTTGAGGTGC | Cloning |
| *OsMPK20-1* | F: GGAGGCCAGTGAATTCATGCAGCAGGATCAGCGCAA | Cloning |
| | R: CGAGCTCGATGGATCCCTAATACATCCTCGACATGC | Cloning |
| *OsMPK20-4* | F: GGAGGCCAGTGAATTCATGGCGATGCAGACGATGCA | Cloning |
| | R: CGAGCTCGATGGATCCCTAATACATCCTCGAGACCC | Cloning |
| *OsMPK7* | F: GGATTCGGAATGACAAGG | qRT-PCR |
| | R: GATCAGCCAACGCCTCTT | qRT-PCR |
| *OsMPK9* | F: CAGTGATGAGTTCAGGGTA | qRT-PCR |
| | R: ACTTTGCTGTTCGGATAA | qRT-PCR |
| *OsMPK20-1* | F: GGTAAGGGAAGCTATGGG | qRT-PCR |
| | R: CTCCTCGATGGAGGCAAC | qRT-PCR |
| *OsMPK20-4* | F: GTGCCCTTGACAACTTTC | qRT-PCR |
| | R: CCCTCGTCCTGTTCTACC | qRT-PCR |
| *OsWRKY55*-UTR-F | F: GATGCAGGAAGATTTGGT | qRT-PCR |
| *OsWRKY55*-UTR-R | R: CTTTGAGGGTGTAGAGGA | qRT-PCR |
